# Identifying hotspots for antibiotic resistance emergence and selection, and elucidating pathways to human exposure: Application of a systems-thinking approach to aquaculture systems

**DOI:** 10.1016/j.scitotenv.2019.06.134

**Published:** 2019-10-15

**Authors:** Lucy A. Brunton, Andrew P. Desbois, Maria Garza, Barbara Wieland, Chadag Vishnumurthy Mohan, Barbara Häsler, Clarence C. Tam, Phuc Nguyen Thien Le, Nguyen Thanh Phuong, Phan Thi Van, Hung Nguyen-Viet, Mahmoud M. Eltholth, Dang Kim Pham, Phuc Pham Duc, Nguyen Tuong Linh, Karl M. Rich, Ana L.P. Mateus, Md. Ahasanul Hoque, Abdul Ahad, Mohammed Nurul Absar Khan, Alexandra Adams, Javier Guitian

**Affiliations:** a Veterinary Epidemiology, Economics and Public Health Group, Royal Veterinary College, Hawkshead Lane, Hatfield AL9 7TA, UK; b Institute of Aquaculture, Pathfoot Building, University of Stirling, Stirling FK9 4LA, UK; c International Livestock Research Institute, P.O. Box 5689, Addis Ababa, Ethiopia; d WorldFish, Jalan Batu Maung, Batu Maung, 11960 Bayan Lepas, Penang, Malaysia; e London School of Hygiene and Tropical Medicine, Keppel Street, London WC1E 7HT, UK; f National University of Singapore, National University Health System, 1E Kent Ridge Rd, Singapore; g School of Biotechnology, International University - Vietnam National University HCMC, Đông Hoà, Thủ Đức, Ho Chi Minh City, Viet Nam; h College of Aquaculture and Fisheries, Can Tho University, Campus 2, 3/2 street, Xuân Khánh, Ninh Kiều, Cần Thơ, Viet Nam; i Research Institute for Aquaculture No. 1, Đình Bảng, Từ Sơn, Bắc Ninh, Viet Nam; j International Livestock Research Institute, 298 Kim Ma Street, Ba Dinh District, Hanoi, Viet Nam; k Faculty of Veterinary Medicine, Kafrelsheikh University, El Guish St., Kafr El Sheikh, Egypt; l Vietnam National University of Agriculture, Trau Quy, Gia Lam, Hanoi, Viet Nam; m Hanoi University of Public Health, 1A Đức Thắng, Phường Đức Thắng, Đông Ngạc, Bắc Từ Liêm, Hà Nội, Viet Nam; n Chattogram Veterinary and Animal Sciences University, Zakir Hossain Road, Khulshi, Chittagong, Bangladesh

**Keywords:** Antimicrobial resistance (AMR), Cá Tra, Mekong Delta, One Health, *Pangasianodon hypophthalmus*, *Penaeus vannamei*

## Abstract

Aquaculture systems are highly complex, dynamic and interconnected systems influenced by environmental, biological, cultural, socio-economic and human behavioural factors. Intensification of aquaculture production is likely to drive indiscriminate use of antibiotics to treat or prevent disease and increase productivity, often to compensate for management and husbandry deficiencies. Surveillance or monitoring of antibiotic usage (ABU) and antibiotic resistance (ABR) is often lacking or absent. Consequently, there are knowledge gaps for the risk of ABR emergence and human exposure to ABR in these systems and the wider environment. The aim of this study was to use a systems-thinking approach to map two aquaculture systems in Vietnam – striped catfish and white-leg shrimp – to identify hotspots for emergence and selection of resistance, and human exposure to antibiotics and antibiotic-resistant bacteria. System mapping was conducted by stakeholders at an interdisciplinary workshop in Hanoi, Vietnam during January 2018, and the maps generated were refined until consensus. Thereafter, literature was reviewed to complement and cross-reference information and to validate the final maps. The maps and component interactions with the environment revealed the grow-out phase, where juveniles are cultured to harvest size, to be a key hotspot for emergence of ABR in both systems due to direct and indirect ABU, exposure to water contaminated with antibiotics and antibiotic-resistant bacteria, and duration of this stage. The pathways for human exposure to antibiotics and ABR were characterised as: occupational (on-farm and at different handling points along the value chain), through consumption (bacterial contamination and residues) and by environmental routes. By using systems thinking and mapping by stakeholders to identify hotspots we demonstrate the applicability of an integrated, interdisciplinary approach to characterising ABU in aquaculture. This work provides a foundation to quantify risks at different points, understand interactions between components, and identify stakeholders who can lead and implement change.

## Highlights

• The contribution of aquaculture to antibiotic resistance is not well understood.
• Systems mapping was used for two aquaculture systems in Vietnam.
• Hotspots were identified for the emergence/selection of antibiotic resistance.
• Human exposure points to antibiotics and antibiotic-resistant bacteria were mapped.
• Findings inform risk quantification and identification of stakeholders to effect change.

## Graphical abstract

**Figure:**
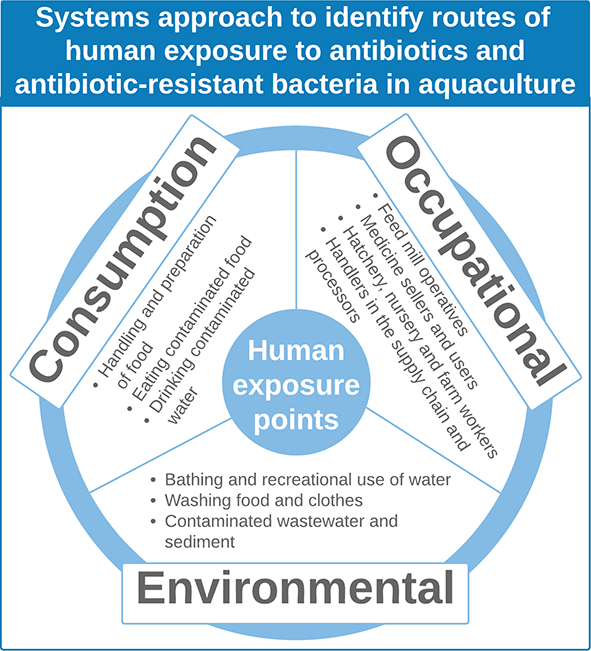

## 1 Introduction

Aquaculture produces more than half of the world's seafood for consumption, and production (tonnage) has grown globally at 6% per year since 2001 (FAO, 2018). Much of this growth is attributable to farms undergoing increasing intensification in low and middle income countries (LMICs), which are now well integrated in the global seafood trade, particularly in Asia (World Bank, 2013; Belton et al., 2018; FAO, 2018). Aquaculture comprises highly complex, dynamic and interconnected systems influenced by environmental, biological, cultural, socio-economic and human behavioural factors. Like other food animal production sectors, aquaculture uses antibiotics not only to combat infectious diseases, but also for prophylactic and growth promotion purposes to help maintain aquaculture stocks (Tuševljak et al., 2013; Pham et al., 2015; Van Boeckel et al., 2015; FAO, 2018; Henriksson et al., 2018; Santos and Ramos, 2018). However, the widespread use of antibiotics in animals and humans has led to the emergence and selection of antimicrobial resistance (AMR).

The tripartite collaboration on AMR between the Food and Agriculture Organization of the United Nations (FAO), the World Organisation for Animal Health (OIE) and the World Health Organization (WHO) recognises the importance of a One Health approach to tackling AMR, and one of the aims of this collaboration is to promote prudent and responsible use of antimicrobial agents (WHO, 2015). While the development of resistance to antibiotics is a natural phenomenon, increasing antibiotic exposure increases selection pressure, and so reducing exposure by limiting total antibiotic usage (ABU) is an important strategy to reduce selection pressure for AMR (O'Neill, 2015).

Little is known about the role that ABU in aquaculture plays in the global problem of antibiotic resistance (ABR). At the greatest levels of intensification, ABU is generally low as the enterprises operating and managing such farming systems have greater resources to implement more effective biosecurity measures, train workers in better husbandry and management practices, and employ other disease prevention measures such as vaccination (Rico et al., 2013; Phu et al., 2016; FAO, 2018). These highly intensified systems often produce for export and typically products have to meet high standards demanded by importing countries; for example, the European Union requires exporting countries to demonstrate adherence to standards covering animal health, hygiene and residues in food (European Union, 2019). Access to export markets provides an important financial incentive to adapt management strategies in production systems to comply with regulations designed to avoid antibiotic residues that may, at least in part, reduce total ABU (Phu et al., 2016; Goutard et al., 2017). At the other end of the spectrum, small-scale farms operating at low stocking densities, and often culturing multiple species in the same system, use low quantities of antibiotics because disease prevalence is typically low. However, much of the growth in global aquaculture output stems from farms transitioning from such small-scale, low intensification systems to systems operating at greater intensification, where the risks of an infectious disease outbreak are greater and farmers often rely on antibiotics to resolve these issues (Rico et al., 2013; Henriksson et al., 2018). Initially, these intensifying farming systems in LMICs generally produce for domestic rather than export markets (Belton et al., 2018).

In aquaculture, antibiotics are usually mixed with feed before administering to animals but drugs may also be applied directly into the aquatic environment (Pham et al., 2015; Okocha et al., 2018). This can lead to the dispersal and leaching of antibiotics into the environment, exposure of both sick and healthy animals and other aquatic organisms to antibiotics, and potentially an increase in the likelihood of human exposure to antibiotics and antibiotic-resistant bacteria (Shen et al., 2018). Extensive and imprudent use of antibiotics in aquaculture can occur in LMICs to treat a myriad of health issues and increase productivity, often to compensate for management and husbandry deficiencies (Van Boeckel et al., 2015; Phu et al., 2016). The lack of diagnostic capacity, vaccines and other effective alternatives to antibiotics (e.g. probiotics) compounds the problem (Henriksson et al., 2018). Enforcement of regulations for the responsible use of antibiotics is often inefficient and surveillance or monitoring of ABU in livestock and aquaculture in many countries is very limited or absent (Cabello, 2006; FAO, 2016; Goutard et al., 2017; Mo et al., 2017; Shah et al., 2017). Where regulations and enforcement do exist, these are largely coordinated by industry and confined to export-oriented commodities, leaving the food animal production systems destined for domestic consumption more vulnerable to inappropriate ABU. There are currently no guidelines on appropriate ABU in relation to the risks of environmental contamination, and a large gap in knowledge exists of the extent to which the environment contributes to ABR in humans (Berendonk et al., 2015; O'Neill, 2015; Thanner et al., 2016; Goutard et al., 2017; Lundborg and Tamhankar, 2017; Bengtsson-Palme et al., 2018).

Aquaculture systems are highly diverse in terms of the production systems used and the species cultured (FAO, 2018), and are often highly interconnected with other food production systems through multiple wide-reaching pathways (Chuah et al., 2016; Watts et al., 2017; Shen et al., 2018). This makes the aquaculture environment particularly vulnerable to the introduction and spread of ABR. A number of studies have demonstrated that resistance can be transferred between fish pathogens, aquatic bacteria and human pathogens (Kruse and Sørum, 1994; Kruse et al., 1995; Rhodes et al., 2000; Molina-Aja et al., 2002; Furushita et al., 2003; Sørum, 2006). Antibiotic resistance genes (ARG) and antibiotic-resistant bacteria, including zoonotic pathogens, have been isolated from water, products and farmers from aquaculture systems across the globe (Cabello et al., 2013; Miranda et al., 2013; Grema et al., 2015; Chuah et al., 2016; Watts et al., 2017; Santos and Ramos, 2018; Shen et al., 2018). However, there is currently little understanding of the human health risks posed by emergence of ABR in aquaculture systems. Moreover, there is a general lack of knowledge and clarity about how aquaculture systems operate and how changes to the drivers of ABU would affect ABR in the system as a whole (Berendonk et al., 2015; Bengtsson-Palme et al., 2018). This reflects a wider failure of existing ABR research to adequately address the challenge from an ecosystems perspective (Berendonk et al., 2015; Hinchcliffe et al., 2018). Using an ecosystems approach to identify the possible human exposure points to antibiotics, antibiotic-resistant bacteria and ARG in aquaculture production is key to reducing human health risks from aquaculture, though data and evidence are lacking in this regard (Miranda et al., 2013; Berendonk et al., 2015; Chuah et al., 2016; Phu et al., 2016; Watts et al., 2017).

Such a thorough understanding of how aquaculture systems operate is required in order to ‘follow’ the actual and potential dissemination of antibiotics, antibiotic-resistant bacteria and ARG throughout the production systems. Complex ecological problems such as ABR cannot be solved by focusing on individual processes (Hinchcliffe et al., 2018); rather, a focus on understanding entire systems is needed in order to identify different components, assess feedback loops and predict behaviours. This requires a systems-thinking approach to describe and understand the complex processes (Hinchcliffe et al., 2018). Systems thinking is the consideration of systems in their totality, as their constituent parts and their interactions, as well as their interaction with the wider environment (Peters, 2014). Systems-thinking approaches, utilising tools such as system dynamics modelling, have been used to understand the behaviour of complex dynamic systems spanning areas such as climate change, environmental policy and disease eradication programmes (BenDor et al., 2018; Kapmeier and Gonçalves, 2018; Tebbens and Thompson, 2018). The participation of a multitude of stakeholders when using a systems approach can maximise stakeholder engagement and ownership of the new knowledge generated, allow for the incorporation of different perspectives on a problem, reveal hidden or undescribed drivers, and encourage networking, interdisciplinarity and systems thinking (Vennix, 1996; Siokou et al., 2014). A recent study showed the potential benefits of this approach in relation to sustainable ABU in cattle farming (Lhermie et al., 2017), but systems thinking is under-used in addressing antibiotic resistance in aquaculture (Hinchcliffe et al., 2018).

The main aim of this study was to apply a participatory systems-thinking approach to map two distinct aquaculture systems and identify potential hotspots for: 1) the emergence and selection of ABR; and 2) human exposure to antibiotics, antibiotic-resistant bacteria and ARG, and to compare potential routes of human exposure to antibiotics in these systems. In addition, we aimed to identify potential drivers of ABU and interventions to reduce ABU through the mapping process.

## 2 Methods

### 2.1 Workshop to map aquaculture systems

A workshop was held with invited experts with experience of the aquaculture sectors in Vietnam and Bangladesh. The workshop had four aims: 1) to develop systems thinking and experience in mapping systems; 2) build collaborations and understanding of different expertise; 3) create maps of the aquaculture sector and the drivers of ABR; 4) identify the most likely routes of exposure to ABR for humans. The workshop took place over two days (18–19 January 2018) in Hanoi, Vietnam and involved 23 attendees from Vietnam, Bangladesh, international agriculture research organisations and the research team based in the United Kingdom (UK) (Table 1). It was carried out as part of a larger project exploring the contribution of aquaculture to ABR with funding from the UK Medical Research Council. The range of disciplines among attendees included epidemiology, veterinary sciences, microbiology, environmental science, anthropology, economics, politics, public health, pathology, aquatic science, aquaculture and biotechnology. The workshop was a combination of presentations, focus group activities and plenary discussions.

**Table 1:** Organisations represented at the workshop and broad areas of expertise.

Organisation	Expertise
Royal Veterinary College, University of London	Veterinary epidemiology, public health, agri-health and economics
Institute of Aquaculture, University of Stirling	Aquaculture, microbiology and biotechnology
London School of Hygiene and Tropical Medicine	Epidemiology and medical anthropology
International Livestock Research Institute	Veterinary epidemiology, agricultural economics, Eco health and food safety
WorldFish	Aquaculture and aquatic animal health management
Chittagong Veterinary and Animal Sciences University, Bangladesh	Veterinary epidemiology, microbiology, public health and aquatic biotechnology
Research Institute for Aquaculture No. 1, Vietnam	Aquaculture and aquatic animal health
National Institute of Veterinary Research, Vietnam	Veterinary hygiene and food safety
Vietnam National University of Agriculture	Veterinary medicine and animal science
Hanoi University of Public Health	Human medicine, epidemiology, public health and environmental health
Can Tho University	Aquaculture and aquatic animal physiology and health
International University, Vietnam National University, Ho Chi Minh City	Aquaculture and marine biotechnology

After an introduction to the aims of the workshop and presentations from researchers based in Vietnam, Bangladesh and WorldFish, workshop participants were given a brief presentation on systems thinking and presented with an example of previous mapping work in the poultry value chain in Bangladesh. Then a group exercise was used to introduce the participants to the mapping process where each group was asked to draw out a simple system. Participants were given suggestions on how to illustrate different components of the system such as tangible and intangible components, governance systems and relationships, economic factors, actors, infrastructure and environmental factors. The exercise highlighted important aspects of stakeholder mapping such as consideration of boundaries (i.e. edges) with other systems.

### 2.2 Selection of aquaculture systems

Following the introductory activities, attendees were split into three groups balanced to contain a range of disciplines and experience. The groups were asked to identify aquaculture systems that would be of greatest importance to explore, in terms of the potential role of antibiotics within the systems and relevance of the cultured species. The systems selected by consensus were striped catfish (*Pangasianodon hypophthalmus*) and white-leg shrimp (*Penaeus vannamei*) production, both located in the Mekong Delta region of Vietnam, and tilapia farming in Bangladesh (data not presented here). The two Vietnamese systems were considered to be both of major importance to the aquaculture industry and distinct in terms of their disease challenges and from an ecosystem perspective. For example, striped catfish have more bacterial diseases and a freshwater ecology, while white-leg shrimp tend to have more viral diseases and a brackish water ecology.

The striped catfish industry in Vietnam, located mainly in the Mekong Delta, has undergone recent rapid development, expansion and consolidation through vertical integration (Nhu et al., 2016). With 5400 ha of farms, in 2018 Vietnam produced 1.42 million tons of striped catfish, increasing 8.4% compared to 2017, and its export value was 2.26 billion USD (D-Fish, 2019). This important sector employs about 100,000 people in this region of Vietnam (around 0.5% of the workforce) and much of its product is exported to the United States, the European Union, China and elsewhere in Southeast Asia (Holmyard, 2013; Rico et al., 2013; Ministry of Agriculture and Rural Development [MARD], 2015; Vietnam Association of Seafood Exporters and Producers [VASEP], 2017a; Nguyen and Jolly, 2018). This export-orientated industry has transitioned successfully to highly intensive production and has benefited from considerable recent investment in improved infrastructure, good governance, and adherence to national regulations on food safety and environmental protection (Holmyard, 2013; Phu et al., 2016; Nguyen and Jolly, 2018). However, a survey of aquaculture farms across Asia found that the percentage of farms using antibiotics and the number of compounds used per farm was significantly greater on striped catfish farms in Vietnam compared with some other aquaculture systems (Rico et al., 2013), though there was no suggestion or evidence that antibiotics were used inappropriately or that food safety was in any way compromised. Indeed, one characteristic of striped catfish valued most by consumers is the safety of the product, especially with regard to the lack of contamination by antibiotics, as strict monitoring is currently in place for chemical residue concentrations in flesh (including antibiotics) in products destined for export (Little et al., 2012; Nguyen and Jolly, 2018).

The striped catfish industry of Vietnam was deemed suitable as a case study to identify possible routes of human exposure to antibiotics because the highly intensive systems are relatively consistent, and the need for high volumes of water exchange necessary for production means pathogens are introduced continually, making ABU more likely (Phu et al., 2016). Importantly, the recent improvements implemented in striped catfish production in Vietnam provide a blueprint for increasing output from other aquaculture systems. Thus, findings from this system could be applied to improve production systems elsewhere during transition to more intensive practices.

In comparison, white-leg shrimp is cultured in Vietnam for local consumption as well as export markets. Vietnam produced 427,000 tons of white-leg shrimp in 2017, up 8.5% compared to 2016 (VASEP, 2017b), and its export value in 2018 was 2.48 billion USD (D-Fish, 2019). Unlike fish producers, shrimp producers cannot rely upon vaccines for disease prevention because crustaceans lack the necessary adaptive immune response for long-term protection against pathogens (Witteveldt et al., 2004). As such, these producers may rely more heavily on antibiotics to ensure the health of stocks, and effective approaches other than vaccination are required in shrimp culture systems to prevent and treat bacterial diseases.

### 2.3 Mapping of the system by stakeholders

We defined ‘mapping of the system’ as the process by which the key components of the striped catfish and white-leg shrimp systems were identified, and connections between components were made based on the flow of inputs, outputs, information and governance. The methodology used here was based on the Network for the Evaluation of One Health (NEOH) approach, which is rooted in systems thinking (Rüegg et al., 2018). Each group contained two members of the project team who played the roles of facilitator and recorder. The role of the facilitator was to ask questions, draw out the system and reflect the map back to the group, while the role of the recorder was to capture the discussions and seek clarification on points of uncertainty and detail.

Large whiteboards or paper sheets affixed to the wall were used to draw out the maps. Different colours were used to distinguish different components of the system (e.g. governance, pre- and post-harvest activities), and sticky notes were affixed to the drawn maps to highlight potential human exposure pathways and hotspots for the emergence and selection of ABR. Aside from spontaneous mutations that confer resistance to an antibiotic, emergence of ABR also concerns the horizontal transmission of ARG between bacteria species, whereas selection is the enrichment of antibiotic-resistant bacteria containing ARG (typically to the detriment of susceptible bacteria). Among other factors, conditions favouring emergence and selection of ABR are exposure to sub-inhibitory and non-lethal concentrations of antibiotics.

The mapping exercise consisted of several iterations, with maps presented to all participants, discussed and modified where necessary to obtain whole-group consensus. In each iteration, the groups mapped out the structure of the system (e.g. hatcheries, nurseries, grow-out ponds, markets and feed mills), recorded inputs and outputs, described the governance structure and how this interacted with the system, and identified potential hotspots for ABR emergence, and for human exposure to antibiotics, antibiotic-resistant bacteria and ARG. At the end of the workshop, the maps were photographed and translated into digital diagrams using the web-based Lucidchart software (https://www.lucidchart.com/).

### 2.4 Validation of the system maps

After the workshop, the discussion notes captured by the recorders were interrogated to identify key hotspots, drivers of ABU, potential interventions to reduce ABU and key knowledge gaps. A summary of the findings, including the digital maps, was shared with the workshop attendees, and feedback invited, collated and analysed. This acted as a basis for validating the finished output. Peer-reviewed and other published literature were reviewed to complement and cross-reference the information obtained in the workshop activities and serve for triangulation purposes. Feedback from attendees, cross-referencing of literature and discussions with colleagues, other subject experts and selected stakeholders were used to clarify areas of uncertainty and produce the final maps. Ultimately, validation of the maps and information extracted from these was achieved through the group of experts that attended the workshop reaching consensus after circulation of final versions of maps for final comments and agreement.

## 3 Results

### 3.1 Mapping of the systems by stakeholders

**Fig. 1:**
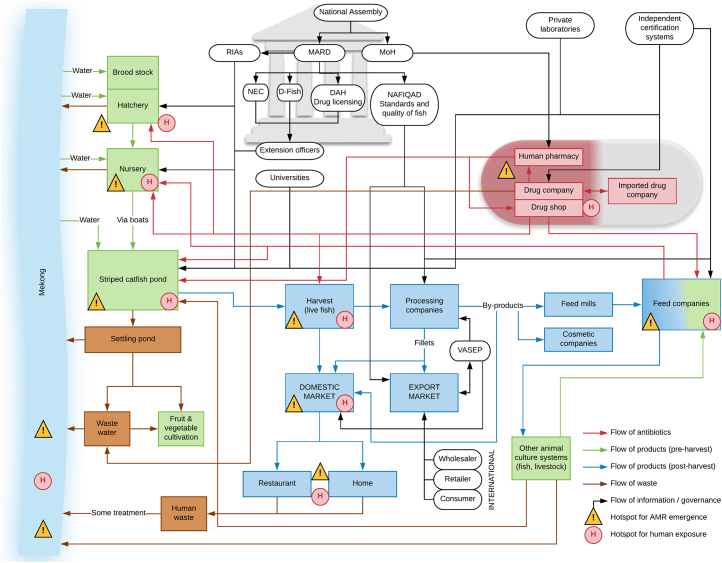
Map of the striped catfish production system in the Mekong Delta, Vietnam. RIAs (Research Institute for Aquaculture 1, 2 & 3), MARD (Ministry of Agriculture and Rural Development), MoH (Ministry of Health), NEC (National Extension Center), D-Fish (Directorate of Fisheries), DAH (Department of Animal Health), NAFIQAD (National Agro-Forestry-Fisheries Quality Assurance Department), VASEP (Vietnam Association of Seafood Exporters and Producers).

**Fig. 2:**
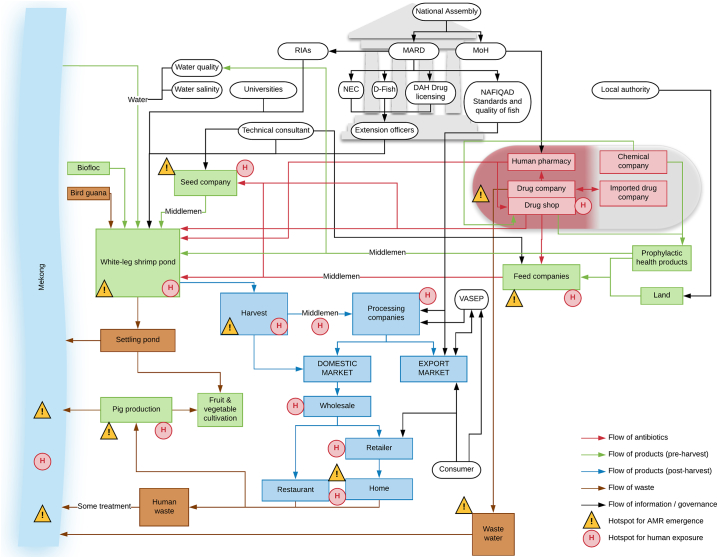
Map of the white-leg shrimp production system in the Mekong Delta, Vietnam. RIAs (Research Institute for Aquaculture 1, 2 & 3), MARD (Ministry of Agriculture and Rural Development), MoH (Ministry of Health), NEC (National Extension Center), D-Fish (Directorate of Fisheries), DAH (Department of Animal Health), NAFIQAD (National Agro-Forestry-Fisheries Quality Assurance Department), VASEP (Vietnam Association of Seafood Exporters and Producers); prophylactic health products refer to a range of feed supplements often added to feed such as prebiotics and probiotics.

The final consensus maps for the striped catfish and white-leg shrimp production systems are presented in Fig. 1, Fig. 2, respectively. The various components of each system map and their flow or connection were grouped broadly into categories, specifically: 1) the environment within which organisms are cultured and other pre-harvest activities (in green); 2) the production, supply and use of antibiotics (in red); 3) activities during and post-harvest including transportation, product sales and consumption (in blue); 4) waste products and their fate (in brown); 5) the network of governance organisations and flow of information (in black). Each system included an intricate set of structures involved in governance of the system and provided an overview of other elements of the system which could be important points for interventions to reduce ABU and ABR, but may not have been identified using a traditional linear thinking approach to follow the flow of antibiotics. For example, improvements to water quality could lead to a reduction in the burden of disease, and therefore to a decrease in the use of antibiotics and disinfectants in the culture systems.

In both maps, the Mekong River was a key boundary to the system. The boundaries ranged from the inputs of water from the Mekong River, feed, drugs, and products from other culture systems, to the outputs of waste products back in to the Mekong River. The maps did not extend beyond these boundaries to consider or map components of wider systems associated with: 1) delivering certain inputs required for culture (i.e. seed, broodstock and biofloc; production of components used in feed; and other systems that input or use the water source); 2) the post-harvest consumption of products by people at home or in restaurants, or after export; 3) waste products and their disposal (e.g. waste products used in other types of livestock or crop production); 4) energy generation and consumption.

The maps illustrate broad similarities between the two systems particularly in the flows of materials needed for production, antibiotics, harvested products and waste. In both systems, antibiotics were deemed most likely to be used during the grow-out phase through application of medicated feeds and during culture at early life stages (hatchery and nursery in striped catfish and seed company in white-leg shrimp system) through addition to water or inclusion in feeds. Medicated feeds are manufactured at the feed mills but shrimp farmers may also prepare their own antibiotic feeds. Antibiotics are sourced generally from drug shops, though human pharmacies represent a possible alternative source (Chi et al., 2017). It was suggested that occasionally antibiotics may be incorporated into the water containing animals after harvest to keep products fresh or to prevent mortality during transport to live markets, but only a fraction of striped catfish production is sold through these markets.

Some key differences were observed between the striped catfish and white-leg shrimp systems. In the white-leg shrimp system, more ‘middlemen’ intermediaries are involved, which reflects the lesser vertical integration and consolidation of this sector compared to the striped catfish system (Fig. 2). This introduces greater variability in production practices and standards within the shrimp value chain; hence, products are sold into a diverse domestic market as well as exported, whereas striped catfish is produced overwhelmingly for export. Some of these middlemen provide farmers with the provisions needed to farm the shrimp via a franchise-style model, including seed and feed, and typically they will purchase the harvested product. The white-leg shrimp farmers are more likely than striped catfish counterparts to use prophylactic health products such as probiotics, though these may be used to condition the culture water at early life stages of both systems. Interestingly, at post-harvest stage in the striped catfish system, by-products are incorporated into the production of feed for other culture systems and these may ultimately feedback into the striped catfish system too (Fig. 1). This represents a point of cross over and interconnectivity between the striped catfish and white-leg shrimp systems, where by-products might be used in the production of feed for shrimp.

### 3.2 Governance

Broadly, the formal structures of governance were similar between the striped catfish and white-leg shrimp systems (Fig. 1, Fig. 2). Participants described the role of the National Assembly in creating veterinary laws and enforcing legislation across both systems. Within the government agencies and departments, the three Research Institutes for Aquaculture (RIAs 1, 2 and 3 based in three regions of Vietnam) were identified as important sources of technical advice at the national level. MARD is in charge of regulations and coordination, and oversees the Department of Animal Health (DAH), Directorate of Fisheries (D-Fish) and National Extension Center (NEC) who are in charge of producing guidelines. Workshop participants indicated that there is flow of knowledge between farmers and government agencies, typically via extension officers. Government extension officers provide information and advice to farmers in both systems to improve production practices, while also conducting surveillance, audits and inspections. Meanwhile, private technical consultants and laboratories, and universities, are further sources of advice, information and support for producers. Under the governance of MARD is the National Agro-Forestry-Fisheries Quality Assurance Department (NAFIQAD) that assesses the standards and the quality of fish products. This department is particularly important in the striped catfish system as they provide testing and certification for producers and processing companies wanting to export. This includes testing for chemical residues in the products as required by the importing countries.

MARD has regional and/or provincial level offices that are responsible for implementing guidelines. NEC is responsible for providing husbandry advice to farmers and promoting the sector. DAH is responsible for veterinary drug licensing (both imported and domestically produced drugs) and inspection of drug usage, as well as disease monitoring. DAH has legal powers to issue fines when the use of forbidden antibiotics is detected, but the frequency of such enforcement (i.e. number of inspections/fines issued) is not known. Participants considered there to be a good relationship between farmers and inspectors. D-Fish provides technical knowledge and may sometimes advise on ABU. A non-governmental organisation identified as providing governance within the systems was VASEP, and their role is to help companies in Vietnam to identify new markets and to promote products overseas. The role of local government in land use and planning for aquaculture sites was discussed as a further authority with potential power to influence acceptability of locations for farming.

Outside of formal governance, the roles of farmers, feed and drug companies were discussed. Many farmer cooperatives exist, but these do not always have a formal structure; however, these networks provide a platform for farmers to help each other, share knowledge, and manage themselves, although they do sometimes work closely together with extension officers. Feed companies provide technical support to various actors in the production chain. Drug companies distribute drugs to drug shops and other retailers for further use, and sometimes supply directly to farmers. Drug shops also provide technical support to various actors in the value chain. There was a perception that different drug companies and drug shops may actively promote ABU as this generates profits. As well as producing drugs domestically, drug companies can import medicines. Participants reported that sometimes drugs are imported directly by drug sellers, that illegal importations may occur, and that illegally imported antibiotics can be supplied to farmers. Furthermore, consumers and importers may exert pressure on producers in aspects of product quality, often via VASEP, through introduction of standards and instruments such as certification schemes, which can influence various actors including farmers, feed companies and processors.

### 3.3 Identification of hotspots

Two types of hotspot were identified on the maps: 1) hotspots where conditions favour the emergence and selection of ABR; 2) points of human exposure to antibiotics and antibiotic-resistant bacteria (Fig. 1, Fig. 2). In general, these different types of hotspot were co-located and found in the striped catfish and white-leg shrimp systems at similar points. Important hotspots for the emergence and selection of ABR included at the early and grow-out phases of production and in the Mekong River, and points where antibiotics inadvertently exert selection pressures on the environmental, animal or human microbiota (Table 2). The grow-out phase, where juveniles are grown to harvest size, was identified as the most likely hotspot for emergence of resistance in both systems due to the animals spending the greatest time at this stage in the production cycle (3 months for white-leg shrimp [Hai et al., 2015]; 7 months for striped catfish [Khoi et al., 2008, Holmyard, 2013]) and the cumulative effect of ABU in the earlier production stages, which may have already selected and enriched for antibiotic-resistant bacteria and ARG. The Mekong River, with waste containing antibiotics discharging from various aquaculture and other activities, was a further key hotspot in the environment where the emergence of ABR may be more likely to occur. Associated with the grow-out phase is the settling pond into which solids and wastewater are drained during harvest. The settling pond is a key point of linkage between systems both as an output, where waste from the settling pond is released into the Mekong, and as an input, where it is used as fertiliser in the cultivation of plant-based foods.

**Table 2:** Putative key hotspots for emergence and selection of antimicrobial resistance genes and bacteria identified from the striped catfish and white-leg shrimp maps and the reasoning underlying their inclusion.

Hotspot	Reasoning
Mekong	Waste contaminated with antibiotic residues and antibiotic-resistant bacteria discharged from aquaculture, agriculture and human activities into environment may encourage emergence of antibiotic resistance and selection of ABR bacteria
Production of early life stages (broodstock, hatchery, nursery, seed company)	Use of medicated feeds and culture water containing antibiotics to prevent or treat disease may encourage emergence of antibiotic resistance and selection of ABR bacteria, particularly if not used correctly
Grow-out stage	Use of medicated feeds containing antibiotics to treat or prevent disease (in large quantities, if used) may encourage emergence of antibiotic resistance and selection of ABR bacteria, particularly if not used correctly Introduction of water (and sometimes waste solids) contaminated with antibiotic residues and ABR bacteria from the environment Animals spend the greatest time at this stage of production and longer exposure increases the likelihood of emergence and selection for antibiotic resistance Cumulative effect of antibiotic use in earlier stages of production may mean bacteria in the system at this stage are enriched already for ABR bacteria and ARG
Harvest	Antibiotics possibly used in liquid during transport of live animals may encourage emergence of antibiotic resistance and selection of ABR bacteria
Consumption	Antibiotic residues in contaminated food may act on the human microbiota to encourage the emergence of antibiotic resistance and selection of ABR bacteria
Other agriculture	Use of water and solid waste contaminated with antibiotic residues and ABR bacteria containing ARG may enter other food production systems
Drug producers and sellers, feed companies	Inappropriate handling and disposal of antibiotics during manufacture of antibiotics and medicated feeds may encourage emergence of antibiotic resistance and selection of ABR bacteria by acting on the human microbiota or bacteria in wastewater
Wastewater	Water contaminated with antibiotic residues discharged from aquaculture, agriculture and human activities may encourage emergence of antibiotic resistance and selection of ABR bacteria

ABR (antibiotic-resistant), ARG (antibiotic resistance genes).

The large number of potential routes of human exposure to antibiotic-resistant bacteria and antibiotic residues that were identified across the two systems can be grouped into three categories: occupational (at the farm and different handling points along the value chain including production and retail), consumption (of food and water contaminated with residues and bacteria) and environmental (Table 3). In general, similar potential routes of exposure were identified for the striped catfish and white-leg shrimp systems, though the latter system appears to have more human exposure risk particularly post-harvest, mainly due to the greater abundance of product destined for domestic consumption. Points identified as potential hotspots for occupational human exposure to antibiotics and antibiotic-resistant bacteria included at hatcheries and nurseries in the striped catfish system (analogous activities are performed by the ‘seed company’ in the white-leg shrimp system) and during the grow-out phases of both systems, where farm workers may prepare and use medicated feeds and be exposed to culture water containing antibiotics (Table 3). Drug shops were identified as human exposure hotspots in both systems where repackaging of antibiotics by drug shop workers into smaller packets is common practice, while feed mill workers may be exposed to antibiotics when manufacturing medicated feeds (Table 3). ‘Middlemen’ in the white-leg shrimp system, transporters of some live striped catfish, as well as workers in processing companies and retailers (particularly for domestic supply), were all identified as being at risk of exposure to antibiotics and antibiotic-resistant bacteria though handling aquaculture products and ABU. Post-harvest processing is different for products destined for domestic consumption and the export market, therefore the risk of exposure varies. For products destined for the domestic market, most processes post-harvest are less likely to be carried out in a standardised and regulated manner, while for products destined for the export market activities are well-regulated and typically use sophisticated processing facilities and procedures to adhere to the appropriate regulations (Phan et al., 2009). In particular, the Mekong River is an environmental hotspot for exposure to antibiotic residues and antibiotic-resistant bacteria because white-leg shrimp, striped catfish and other aquaculture systems release wastewater into the river and take water from it, alongside other agricultural and household activities (Table 3). Recreational or domestic use of the river system may bring people into contact with antibiotic residues, antibiotic-resistant bacteria and ARG. In both systems, consumption of aquatic products contaminated with residues and/or antibiotic-resistant bacteria was deemed to be a potential hotspot for human exposure (Table 3).

**Table 3:** Possible human exposure points to antibiotics and antibiotic-resistant bacteria in striped catfish and white-leg shrimp production systems in Vietnam.

Human exposure point	Antibiotic	ABR bacteria
Occupational		
Seed company operatives (shrimp only)	✓	✓
Hatchery and nursery operatives - preparation of medicated feed (striped catfish only)	✓	✗
Hatchery and nursery operatives - administration of medicated feed (striped catfish only)	✓	✗
Hatchery and nursery operatives - contact with contaminated culture water (striped catfish only)	✓	✓
Hatchery and nursery operatives - contact with contaminated wastewater (striped catfish only)	✓	✓
Hatchery and nursery operatives - contact with contaminated inflow water from Mekong River (striped catfish only)	✓	✓
Hatchery and nursery operatives - removal of contaminated sediment (striped catfish only)	✓	✓
Hatchery and nursery operatives - contact with contaminated product (striped catfish only)	✓	✓
Grow-out farm workers - preparation of medicated feed	✓	✗
Grow-out farm workers - administration of medicated feed	✓	✗
Grow-out farm workers - contact with contaminated culture water	✓	✓
Grow-out farm workers - contact with contaminated wastewater	✓	✓
Grow-out farm workers - contact with contaminated inflow water from Mekong River	✓	✓
Grow-out farm workers - removal of contaminated sediment	✓	✓
Grow-out farm workers - contact with contaminated product	✓	✓
Harvesters - contact with contaminated product	✓	✓
Transporters in the supply chain - contact with contaminated product	✓	✓
Handlers in the supply chain - contact with contaminated product	✓	✓
Processors - preparing feeds, by-products or food	✓	✓
Feed mill workers - preparation of medicated feed or feed with contaminated waste product	✓	✓
Food preparers (chefs and cooks) - contact with contaminated product	✓	✓
Medicine store workers - handling of antibiotics	✓	✗
Fruits and vegetable growers - use of contaminated wastewater and sediment	✓	✓
Environmental		
Contact with contaminated wastewater through bathing and recreational use	✓	✓
Contact with contaminated wastewater through washing clothes	✓	✓
Contact with contaminated wastewater through washing food	✓	✓
Contact with contaminated wastewater through domestic cultivation of fruits and vegetables	✓	✓
Contact with sediment from the system through domestic cultivation of fruits and vegetables	✓	✓
Preparation of contaminated product for consumption	✓	✓
Consumption		
Human consumption of contaminated product	✓	✓
Human consumption of contaminated water	✓	✓
Human consumption of fruits and vegetables cultivated with contaminated water	✓	✓

ABR (antibiotic-resistant).

### 3.4 Drivers of ABU and ABR and potential interventions

Several drivers of ABU were highlighted and discussed (Table 4), and these were characterised by economic factors, which could either be incentives for use (e.g. growth promotion, therapeutic and prophylactic use, and market demand) or disincentives for using alternative approaches (i.e. capital required to invest in better production practices), and by individual and operational factors such as a lack of information at the farm level in terms of diagnosis and the impact of imprudent ABU on ABR.

**Table 4:** Factors driving the use of antibiotics in striped catfish and white-leg shrimp production in the Mekong Delta, Vietnam.

Drivers of antibiotic usage
Economic factors
Lack of affordable and practical alternatives to antibiotics such as vaccines Easy accessibility (availability, cost) to antibiotic products Lack of capital to invest in producing a higher quality product (e.g. costs of certifications) Increased market demand for products
Individual factors
Low awareness of the broader impact of ABU and ABR The influence of other farmers
Operational and governance factors
High disease burden Inadequate diagnostic capacity Low level of effective assistance in the field by extension services to tackle aquatic diseases The influence of pharmaceutical and feed companies Seen as an easier (and lower cost) alternative to good biosecurity and better production management Lack of/poor enforcement of existing regulation on ABU

ABR (antibiotic-resistant), ABU (antibiotic usage).

Drivers of ABR were hypothesised to be the direct use of antibiotics, or indirect use through the utilisation of manure from poultry and other species to enrich the water during the grow-out stage, as well as water and resources polluted with antibiotics or antibiotic-resistant bacteria. In the workshop discussions, some participants stated that the farmers only use antibiotics therapeutically and not to promote growth, and that losses due to outbreaks of disease are the main problem for the industry. However, other participants stated that antibiotics may sometimes be used prophylactically with the aim of improving growth or preventing disease outbreaks. A consensus was not reached, so further information needs to be sought from the relevant stakeholders in the field. Moreover, the value of the stock present at the grow-out stage was suggested to be an incentive for ABU at this stage.

Potential interventions to reduce ABU and ABR in the white-leg shrimp and striped catfish systems were identified during the workshop (Table 5), along with the actor(s) deemed best placed to lead each intervention. Suggested interventions ranged from those targeted at the farm level, to those directed at market level and at consumers. Many of these interventions are likely to require leadership from government and industry, while some that relate to management practices could be led and implemented by individual farmers (Table 5).

**Table 5:** Potential interventions to reduce the use of antibiotics in striped catfish and white-leg shrimp production in the Mekong Delta, Vietnam.

Potential interventions	Suggested stakeholder lead
Improvement of hygiene and biosecurity	Farmer/industry
Use of specific pathogen-free stock	Farmer/industry
Promotion and application of better management practices e.g. Vietnamese Good Agricultural Practices (VietGAP)	Farmer/industry
Development of breeds less susceptible to bacterial diseases	Industry
Formalisation of farmer cooperatives for knowledge sharing	Industry
Improve availability of alternatives to antibiotics, such as vaccines, probiotics, prebiotics, immunostimulants, immunomodulators	Industry/research
Removal of commission structure for drugs sellers and drug company quotas	Government
Design and implementation of disincentives for ABU and incentives to produce antibiotic-free products	Government/industry
Development and application of certification systems for antibiotic-free products and harmonisation of third party existing schemes to improve enforcement	Government/industry
Creation and implementation of ABR awareness campaigns targeted at farmers	Government/industry
Consumer awareness campaigns to encourage smart choices by consumers e.g. Food Clear Association	Government/industry
Development of rapid tools and increased diagnostic capacity in the field	Research/government/industry

ABR (antibiotic-resistant), ABU (antibiotic usage).

### 3.5 Knowledge gaps

A number of knowledge and data gaps became apparent during the workshop. A critical problem is the inadequate data on ABU (quantities, quality, types and purposes) at different points of the aquaculture value chain, particularly at farm level, but also in terms of sales of drugs at the retail and wholesale levels. In addition, there is little information to allow quantification of the risk of exposure at different points in the system, such as the levels of residues or the prevalence of economically important fish and shrimp diseases, zoonotic bacteria and ARG at the different hotspots. This is due to a lack of surveillance meaning that management of disease outbreaks and ABR is currently reactive, and thus similar to the situation observed in many other countries and food production systems (Goutard et al., 2017). In all, this makes it extremely difficult at this time to conduct risk analysis to determine the importance of the multiple potential pathways for exposure to antibiotic-resistant bacteria and residues in the system.

Another important area identified in which knowledge is lacking is on the drivers of human behaviour in relation to ABU, and the potential role and impact of incentives and other interventions designed to achieve changes in behaviour. Once again, this is a knowledge gap not only in aquaculture but across agricultural sectors. Workshop participants were unable to identify any social norms or values that would influence the behaviour of producers; instead, economic drivers were deemed likely to be most influential.

In terms of the structure of the two systems, participants reported a lack of information on how production is related to, and integrated with, other agricultural systems such as pigs, poultry and crop production, and the effect that ABR in aquaculture can have on these other production systems and vice versa. Moreover, government policy at national and regional level is encouraging integration of different production systems through co-cultivation, such as rice and shrimp. While information on these systems is collected and detailed in government reports, this is not always available publicly.

Finally, the participants reported a lack of denominator data for residue failures at testing of products destined for export. Residue testing is performed by NAFIQAD, but information on the number of tests performed is lacking, and typically importing countries will only report the number of shipments of a product that failed their own tests at the border. Further, as testing for residues in aquaculture-produced food for domestic consumption is not performed routinely, few data are available on this subject.

## 4 Discussion

The aim of this study was to map the components, interactions and flow of products in two distinct commercially important aquaculture systems to identify potential hotspots for the emergence and selection of ABR and hotspots for human exposure to antibiotics, antibiotic-resistant bacteria and ARG. Concomitantly, the study sought to identify potential drivers of ABU and interventions to reduce ABU and ABR in the selected systems, specifically striped catfish and white-leg shrimp production in Vietnam.

A systems-thinking approach was taken to address this problem, as it helps to bring together the views of different stakeholders and fosters interactions between subject experts, while combining into the same analytical framework diverse concepts ranging from molecular microbiology to social sciences, thus allowing a comprehensive understanding of the complexities of systems and allowing identification of knowledge gaps (Peters, 2014). Such an approach was necessary to develop a thorough knowledge of how the systems operate, so that the emergence and transmission of ARG and antibiotics could be followed through the production systems. Complex ecological problems such as ABR cannot be solved by focusing on individual processes; rather, a focus on understanding systems in their totality is needed in order to identify different components and their interaction, assess feedback loops and predict behaviours (Goutard et al., 2017; Hinchcliffe et al., 2018).

Maps of components in the striped catfish and white-leg shrimp systems allowed the identification of potential hotspots for the emergence of ABR (which is more likely to occur in the presence of antibiotics) in bacteria, and human exposure to antibiotics and antibiotic-resistant bacteria. In general, similar hotspots were identified across both systems, particularly in terms of the roles of the grow-out stages and the Mekong River. Some differences were observed post-harvest, mainly due to the difference in intended markets. In line with our hotspots, previous studies have detected the presence of antibiotic residues in aquaculture production in Vietnam: Le et al. (2005) detected sulphonamides, quinolones and trimethoprim at black tiger shrimp farms located in the mangroves of Thai Binh Province, Nam Dinh Province, Can Gio district – Ho Chi Minh City, and Ca Mau Province, while Andrieu et al. (2015) detected enrofloxacin up to 680 ng L^−1^ at the wastewater discharge point from striped catfish farms. Further, Giang et al. (2015) reported the presence of at least one antibiotic in 91.6% of 154 surface water samples from areas of the Mekong Delta that receive aquaculture wastewater, while Nakayama et al. (2017) reported the presence of sulphonamides and ARG to sulphonamides and beta-lactams in freshwater and aquaculture sites in Can Tho city. Together, these observations support the grow-out stage and the Mekong River to be critical points for the potential emergence and dissemination of ARG in the environment.

The environment is an often neglected pathway in ABR studies, assessments and policies. One study suggests that a lack of enforcement of legislation means that the disposal of wastewater from aquaculture into the environment is common practice in the Mekong Delta. In addition, the costs of wastewater treatment are greater than the fine imposed for environmental pollution so the incentive for change in the sector is low (Genschick, 2011). There remains a high degree of uncertainty as to the role of the environment as an output (potentially receiving and harbouring ARG and residues from the system and agricultural wastewater) and, simultaneously, as an input into the system through use of this contaminated resource for culture (Berendonk et al., 2015; Berglund, 2015; Chuah et al., 2016; Thanner et al., 2016; Watts et al., 2017). The impact of antibiotics entering the environment is poorly understood (Binh et al., 2018), though the presence of antibiotics in wastewater discharged into the Mekong River is associated with reductions in bacterial diversity (Nakayama et al., 2017). Effects on bacterial communities may have important implications for the functioning of environmental ecosystems and the emergence of ABR, with the impacts of this yet to be understood (Berendonk et al., 2015; Thanner et al., 2016; Watts et al., 2017).

Industry consolidation in aquaculture in Vietnam has been driven by international trade requirements (Nguyen and Jolly, 2018), and this has created opportunities for entrepreneurs to meet the needs of the domestic consumers for affordable aquatic protein by producing animals more cheaply, as well as culturing alternative species (Belton et al., 2018). This divergence in producing for export and domestic markets is observed in the shrimp sector in Vietnam. Consumer perception can drive improvements in farming practices with respect to ABU and this is a potentially powerful force that can be exerted by consumers, either in-country or in the major export territories (Holmyard, 2013; Rico et al., 2013). Though this pressure may not be as intense in domestic production, it may be an important force to harness in future to encourage practice improvements, especially as aquatic products represent a considerable proportion of the diet in Vietnam (Pham et al., 2015). The importance of aquatic food is likely to increase and, along with it, so might the influence of the domestic consumer. Further research is needed to explore how the information gathered about the system and stakeholders in this present study could support an ex-ante assessment on how shaping consumer demand will impact the structure and operation of the system.

A key question remains around the introduction of improved practices: will there always be a market for cheaper products produced to lower standards? This may be so, but there is a clear need to move towards more sustainable animal protein production to meet the demands of an expanding human population (FAO, 2018). The increasing intensification of production for domestic consumption perhaps paves the way to increased consolidation in future domestic supply (Belton et al., 2018). Such industrial consolidation of products for local consumption might deliver improved production practices that, in turn, could reduce reliance on antibiotics. However, there likely will still be a need for economic incentives or enforceable regulation on the use of antibiotics in this market to ensure prudent ABU and an effective reduction in total ABU (O'Neill, 2015; Henriksson et al., 2018). In addition, it is the farms transitioning to intensive production that are most likely to require antibiotics to maintain production as culture intensity increases, but the ability to invest capital in improved biosecurity and disease prevention measures has yet to be realised through increased profitability (Nguyen and Ford, 2010; Rico et al., 2013). Support could be offered through temporary subsidies or attractive loans to producers who aim to change their production system.

The majority of potential interventions to reduce ABU and ABR identified at the workshop require leadership and support from government and industry. While the roles of government agencies were clearly defined at the workshop, the role of industry leaders was not so apparent. It is crucial that industry is engaged to provide leadership and support for interventions to reduce ABR. The feasibility and effectiveness of each intervention identified will vary according to the production system. The consolidation of the striped catfish sector to farm high-quality products for export has meant that the sector has been able to invest in improved biosecurity and disease prevention measures, which includes better training of farm workers, preparation of standard operating procedures and improved record keeping (Holmyard, 2013; Phu et al., 2016; Chuah et al., 2016). Certification of aquaculture products for securing international trade has been an important driver to improve biosecurity, farm management and animal husbandry practices, including responsible ABU, thus proving that this powerful incentive can improve farming practices (FAO, 2011; Henriksson et al., 2018). The resources for such investment in farming practice improvements are less readily available to farmers and organisations that may be producing for domestic consumption, as these generally produce smaller volumes of products for lower profit (Belton et al., 2018; FAO, 2018). Smaller farms are more likely to lack robust biosecurity and therefore might be more vulnerable to epizootic events when intensifying production, and consequently may rely more heavily on antibiotics to protect animal health as a cost effective option (Rico et al., 2013; Phu et al., 2016). Some interventions to reduce ABU and ABR are aimed at addressing farmer knowledge of best practice and awareness of ABR. Pham et al. (2015) found that only 16% of farmers knew about regulations relating to ABU in aquaculture. However, in a recent survey, aquaculture producers had significantly (*p* < 0.01) better knowledge of ABR compared to pig and poultry producers in Vietnam (Phuc et al., submitted). Both studies suggested that farmers tended to rely on information on ABU and ABR from drug sellers and drug companies (Pham et al., 2015; Phuc et al., submitted), and this is consistent with our present study, although other information providers ranging from government extension officers to feed companies to farmer cooperatives were also identified. There is a conflict of interest in the role of drug sellers as information providers because there is a financial incentive for drug companies to promote the use of their antibiotics to farmers, with drug sellers usually paid by commission. The provision of information by local government extension officers could help improve practices by farmers (Phu et al., 2016); however, Pham et al. (2015) reported that only 32% of farms surveyed were inspected by officials and this was usually only to assess water quality. Strengthening communication between farmers and government extension officers might offer an opportunity to reduce inappropriate ABU and ABR.

A key knowledge gap identified by workshop participants was the lack of information on ABU at different points of the systems. A study of various aquaculture systems across Asia found that all 17 striped catfish farmers surveyed in Vietnam used antibiotics (17 different antibiotics belonging to 10 classes) (Rico et al., 2013), while another reported that farmers in northern Vietnam used antibiotic tablets sold for human use to treat fish (Chi et al., 2017). Moreover, fluoroquinolones, which have been banned from use in aquaculture in Vietnam since 2009, are still reportedly used in striped catfish and white-leg shrimp farming (Andrieu et al., 2015; Chi et al., 2017). Phu et al. (2016) found that striped catfish farmers in Vietnam reported using 24 antimicrobials, and Rico et al. (2013) calculated that 93 g of antibiotics were used per tonne of harvested fish, which is actually lower than estimated for other meat production sectors such as pigs and chickens (Van Boeckel et al., 2015). A recent review by Binh et al. (2018) summarised knowledge of ABU and ABR from existing independent surveys, and highlighted the lack of structured monitoring and surveillance in the AMR National Action Plan of Vietnam. Binh et al. (2018) reported that >30 antibiotics are permitted for use in aquaculture in Vietnam, including those described as critically important antimicrobials for human medicine by the WHO (2017), and the authors concluded that determining the prevalence of antibiotics in the aquatic environment is essential for the success of the National Action Plan of Vietnam.

Another knowledge gap identified in this present study was how aquaculture production is integrated with other agricultural food production systems. Integrated agriculture-aquaculture systems, which are sustained by the addition of waste from livestock and human sources, have been encouraged in Vietnam due to their claimed economic efficiencies (Hai et al., 2015). For example, integrated shrimp farming and rice farming accounted for 40,350 ha of the farming area in Ca Mau province in 2013, with a harvest size of 20–40 individuals/kg. It generated the second highest annual production yield among different types of agricultural production with 475.5 kg/ha/year (Department of Agriculture and Rural Development, 2013). While such integrated farming may be economically beneficial, it may also pose a risk for contamination of the aquatic environment with antibiotics, antibiotic-resistant bacteria and ARG from livestock and plant-based agriculture (Hoa et al., 2010; Hoa et al., 2011; Watts et al., 2017).

Many farmers work with low margins so every dollar invested has to yield profit. Often alternatives are not perceived to be as effective as antibiotics, and even if individual interventions are low cost, the upfront outlay can be prohibitive. Vaccines, for example, may help to prevent disease but are often pathogen-specific, and so a farmer might need to invest in several while just one antibiotic may be used to treat a range of bacterial diseases (Phu et al., 2016; Henriksson et al., 2018). As identified in this present study, the key hotspots for human exposure to antibiotics in white-leg shrimp and striped catfish culture are largely similar; however, the solutions that will lead to reduction and elimination of these human exposure points will likely be distinct between the systems. Though vaccines may be effective to reduce ABU in striped catfish culture if they are available and used correctly, alternatives (such as biofloc and pre- and probiotics) may be more effective in white-leg shrimp culture because these organisms do not possess adaptive immunity on which vaccination relies (Witteveldt et al., 2004). Other solutions not indicated on the maps but that may reduce or eliminate ABU would be the development of breeds that naturally resist bacterial pathogens (Cock et al., 2009; Moss et al., 2012).

One limitation of this present study was the lack of industry (producers and input providers such as feed and drug sellers) and consumer-group representation at the workshop, as well as attendees from other governmental agencies. These stakeholders would have provided valuable additional insights into the structure and risk points in the systems, and helped to support some of the observations. It may be that alternative approaches such as interviews would be better suited to engage these stakeholders, and these will be incorporated into follow up activities. Furthermore, in this present study, we focused on two aquaculture systems in Vietnam and as such our findings are context specific. However, many of the observations, findings and conclusions can be generalised and applied to other systems with similar characteristics in different locations. Indeed, it is likely that bespoke strategies will need to be developed for different territories and sectors (O'Neill, 2015).

### 4.1 Conclusion

By using systems thinking and mapping by stakeholders to identify hotspots we have demonstrated the applicability of an integrated, interdisciplinary approach to characterising ABU in aquaculture in order to understand fully the consequences of ABU, how this relates to the emergence and spread of ABR, and ultimately its public health impacts. This work provides a platform to quantify risks at different points, understand interactions between components, and identify key stakeholders who can lead and implement change. The maps generated from this participatory approach can be used to understand the flow of antibiotics, antibiotic-resistant bacteria and ARG in the system and they offer a basis for identifying points where an intervention may reduce ABU, antibiotic-resistant bacteria and ARG, while allowing evaluation of the effectiveness of interventions through identifying points that best allow quantitative monitoring (i.e. through surveillance measures) of these risks.
